# Gamete Nuclear Migration in Animals and Plants

**DOI:** 10.3389/fpls.2019.00517

**Published:** 2019-04-24

**Authors:** Umma Fatema, Mohammad F. Ali, Zheng Hu, Anthony J. Clark, Tomokazu Kawashima

**Affiliations:** ^1^Department of Plant and Soil Sciences, University of Kentucky, Lexington, KY, United States; ^2^The Key Lab of Plant Pathology of Hubei Province, Huazhong Agricultural University, Wuhan, China

**Keywords:** fertilization, gamete nuclear migration, F-actin, microtubule, sexual reproduction, cytoskeleton

## Abstract

The migration of male and female gamete nuclei to each other in the fertilized egg is a prerequisite for the blending of genetic materials and the initiation of the next generation. Interestingly, many differences have been found in the mechanism of gamete nuclear movement among animals and plants. Female to male gamete nuclear movement in animals and brown algae relies on microtubules. By contrast, in flowering plants, the male gamete nucleus is carried to the female gamete nucleus by the filamentous actin cytoskeleton. As techniques have developed from light, electron, fluorescence, immunofluorescence, and confocal microscopy to live-cell time-lapse imaging using fluorescently labeled proteins, details of these differences in gamete nuclear migration have emerged in a wide range of eukaryotes. Especially, gamete nuclear migration in flowering plants such as *Arabidopsis thaliana*, rice, maize, and tobacco has been further investigated, and showed high conservation of the mechanism, yet, with differences among these species. Here, with an emphasis on recent developments in flowering plants, we survey gamete nuclear migration in different eukaryotic groups and highlight the differences and similarities among species.

## Introduction

Each generation of sexually reproducing species depends on the blending of maternal and paternal genomes by fertilization. For that fusion of female and male gamete nuclei (karyogamy) to occur, female and male gamete nuclei must migrate toward each other in the fertilized egg (the zygote). In some animals such as mice, the blending of parental genomes takes place in the two-cell embryo after the completion of the first zygotic division (Reichmann et al., 2018). Yet, the migration of both gamete nuclei to be proximal to each other in the mouse zygote prior to the first division is also essential for the successful initiation of the next generation. In animals, unlike plants, the completion of halted meiosis of the maternal genome takes place in the zygote after fertilization but before the fusion of parental genomes. The exchange of male gamete chromatin proteins from protamines to histones also occurs during gamete nuclear migration in animals and early-diverging land plants (Reynolds and Wolfe, 1984). Taken together, gamete nuclear migration plays a pivotal role in successful fertilization regardless of whether fertilization involves karyogamy or non-karyogamy or is in animals or plants and thus it is important to understand how organisms control such essential cellular dynamics.

**FIGURE 1:**
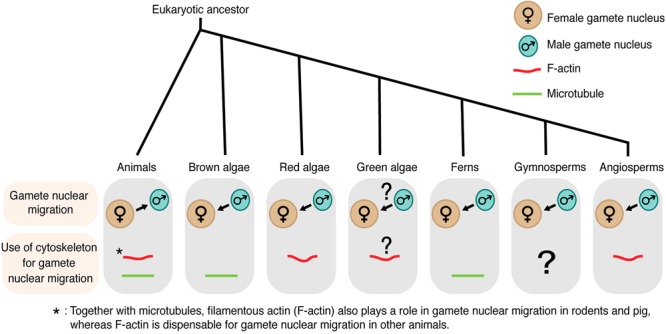
Gamete nuclear migration and use of cytoskeleton for gamete nuclear migration in different kingdoms. In most animals, microtubules assist the migration of both male and female gamete nuclei toward their fusion site. Unlike animals, male gamete nucleus migrates to female gamete nucleus for fusion in algae and land plants. The direction of gamete nuclear migration in green algae is unclear. The use of cytoskeleton also varies among different groups within the same kingdom. In case of algal group, brown algae use microtubule for gamete nuclear migration, whereas red algae use F-actin. F-actin plays a role in green algae (*Chlamydomonas*) mating but its involvement in gamete nuclear migration is not clear. Among land plants, ferns use microtubule, whereas angiosperms use F-actin for sperm nuclear migration. The arrow shows the overall direction of the nuclear movement.

Since the discovery of sperm in semen in the late 1600s, the knowledge of gamete nuclear migration during fertilization has been first advanced in animals, including the first observation of male and female gamete nuclei fusion in sea urchin by Oskar Hertwig in the mid-1800s (reviewed in Birkhead and Montgomerie, 2009). With advances in microscopy, we have a handful of information about how gamete nuclear migration is controlled in many organisms including flowering plants. Cytoskeleton inhibitors have widely been applied and their effects on gamete nuclear migration and fertilization have been analyzed. Tools of molecular biology and live-cell imaging also made it possible to examine and dissect mechanisms underlying gamete nuclear migration. Interestingly, there are large differences in the modes of gamete nuclear migration among organisms (Figure 1), while their goal, to complete fertilization, is the same. What do we know about gamete nuclear migration? To what extent are the cytoskeletons [the microtubule and/or filamentous actin (F-actin)] involved? In this review, we focus on new discoveries in flowering plants and place them in the context of what is known about gamete nuclear migration from animals to algae to early-diverging land plants.

In animals, gamete nuclei during the process of fertilization are termed pronuclei. By contrast, the term pronucleus is hardly employed in plants. This is because unlike animals, meiosis is already completed in the gametes of plants before fertilization. In flowering plants, the cell cycle phase of gametes is estimated between G_1_ and G_2_ (Friedman, 1999). The cell cycle of both male and female gamete nuclei must synchronize for karyogamy [G_1_ in monocotyledonous plants such as barley (Mogensen and Holm, 1995), maize (Mogensen et al., 1995), and rice (Sukawa and Okamoto, 2018), and G_2_ in *Arabidopsis thaliana* (Friedman, 1999)]. Hence, in this review, we name all haploid gamete nuclei simply as nuclei irrespective of if they belong to animals or plants.

## Microtubules Play a Pivotal Role in Animal Gamete Nuclear Migration

**FIGURE 2:**
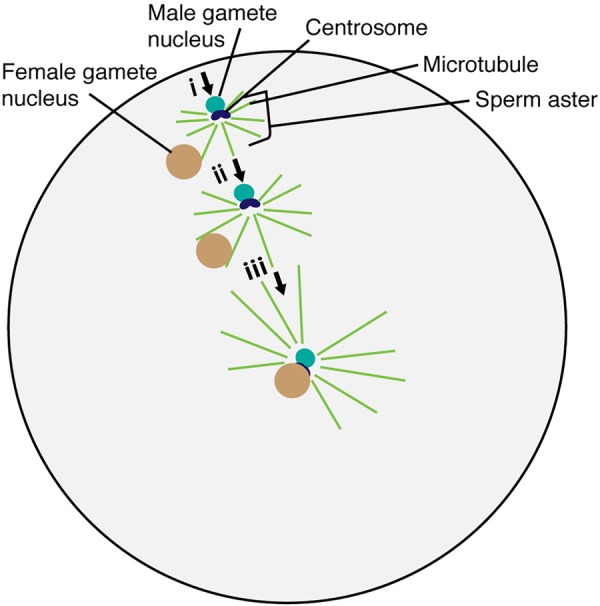
Migration and fusion of egg and sperm nuclei in the sea urchin egg cytoplasm. The sperm aster forms around the base of the sperm head after incorporation into the egg cell. Astral microtubule interacts with the female gamete nucleus (i). Astral microtubules increase in length, pushing the male gamete nucleus and pulling the female gamete nucleus for fusion (ii). The complex then moves to the center of the egg to complete the fertilization process (iii).

Detailed observation of gamete nuclear migration was first performed in the sea urchin, *Lytechinus variegatus.* Using light microscopy, Chambers (1939) discovered that sperm asters were generated around the sperm nucleus during migration (Figure 2). Longo and Anderson (1968) utilized electron microscopy in the sea urchin, *Arbacia punctulata*, and visualized essential ultrastructural components of the sperm aster including the centrioles. Two centrioles and pericentriolar materials around them constitute the centrosome that serves as the microtubule organizing center, generating radially arrayed microtubules around the sperm nucleus.

The sperm aster was also discovered in other invertebrates such as the Atlantic surf clam, *Spisula solidissima* (Longo and Anderson, 1970) and the blue mussel, *Mytilus edulis* (Longo and Anderson, 1969). Using immunocytochemistry and laser scanning confocal microscopy, the organized microtubules of the sperm aster have also been visualized in fruit fly *Drosophila melanogaster* (Callaini and Riparbelli, 1996), nematode *Caenorhabditis elegans* (Strome and Wood, 1983), zebrafish, *Danio rerio* (Solnica-Krezel and Driever, 1994) and even in mammals such cow, *Bos taurus* (Navara et al., 1994), goat, *Capra aegagrus hircus* (Velilla et al., 2005), pig, *Sus scrofa domesticus* (Kim et al., 1997), and rabbit, *Oryctolagus cuniculus* (Longo, 1976). The application of microtubule inhibitors prevented the formation and function of the sperm aster (Supplementary Table 1) and nuclei failed to fuse in most of these afore-mentioned animals (Zimmerman and Zimmerman, 1967; Aronson, 1971; Bestor and Schatten, 1981; Schatten and Schatten, 1981; Strome and Wood, 1983; Schatten et al., 1985; Kim et al., 1996, 1997). Deletion of a centrosomal protein encoding gene *SPD5-1* resulted in the failure of gamete nuclear migration in *C. elegans* (Hamill et al., 2002), confirming that microtubules are key to generating the force for gamete nuclear migration in animals.

## The Origin of Microtubule Arrays for Gamete Nuclear Migration Varies Among Animals

In oogamous reproduction (a large sessile egg and small motile sperm), the paternal contribution to the cellular processes within the fertilized egg was thought to be minimal. However, Bestor and Schatten (1981) performed immunofluorescence against tubulin in sea urchins *A. punctulata* and *L. variegatus* and discovered that the unfertilized egg did not contain microtubules until the sperm nucleus was incorporated. After incorporation, the organized microtubules of the sperm aster formed around the base of the sperm head, containing the centrosome (Figure 2). Astral microtubules increased in length and number over time, pushing the male nucleus from the gamete fusion site into the nuclear fusion site of the egg cytoplasm in sea urchins (Figure 2) (Allen, 1954). The egg nucleus started to migrate only when the astral fibers extend to the periphery of the egg nucleus (Chambers, 1939). These results suggest that the paternally inherited centrosome is the center for microtubule organization and controls gamete nuclear migration during fertilization in animals.

Using anti-tubulin immunofluorescence microscopy, the inheritance of paternal centrosomal materials and its importance in gamete nuclear migration have been validated for *D. melanogaster* (Callaini and Riparbelli, 1996), human *Homo sapiens* (Sathananthan et al., 1991), sheep *Ovis aries* (Le Guen and Crozet, 1989; Crozet, 1990), and rhesus monkeys *Macaca mulatta* (Wu et al., 1996). The proximal sperm centriole was found throughout sperm maturation, during its incorporation into the cytoplasm of the egg and even after fertilization at the spindle poles of the fertilized embryo (Crozet, 1990; Sathananthan et al., 1991). Interestingly, Schatten et al. (1986) could not detect any centrosomal antigen from the sperm but detected it in the egg cell of mouse, *Mus musculus*, oocytes during fertilization, indicating that rodent’s control of gamete nuclear migration underwent a distinct evolutionary path.

## F-Actin Also Plays a Role in Animal Gamete Nuclear Migration

Besides the inheritance of the centrosome, are there any other differences in the control of gamete nuclear migration among animals? Changes in distribution of another cytoskeleton component, F-actin, during the formation and migration of mouse gamete nuclei, were viewed by anti-actin immunofluorescence microscopy (Maro et al., 1984). After fertilization, the homogeneous F-actin meshwork in the mature mouse oocyte generated denser meshwork between each gamete nucleus and the proximal cell cortex. Application of F-actin inhibitor, cytochalasin D prevented sperm nuclear migration in mouse (Maro et al., 1984) and treatment with cytochalasin B inhibited the movement of both male and female pig gamete nuclei during *in vitro* fertilization (Kim et al., 1996). However, the application of F-actin inhibitor did not affect gamete nuclear movement in cow (Sutovsky et al., 1996), sea urchin (Longo, 1980), sheep (Le Guen et al., 1989) and zebrafish (Wolenski and Hart, 1988) (Supplementary Table 1). Together with no inheritance of paternal centrosomal components in rodents, the essential role of F-actin in mouse gamete nuclear migration shows a distinct mechanism evolved specifically in rodents.

Not only the involvement of F-actin itself, but also the expression of the dominant-negative form of Myosin-Vb (Myosin-Vb tail) in the fertilized mouse resulted in defective gamete nuclear migration (Chaigne et al., 2016). The actin nucleator, Formin 2, was also found to be involved in the formation and dynamics of a cytoplasmic mesh of F-actin in the mouse egg for the egg nucleus positioning to the center (reviewed in Almonacid et al., 2018). Xiong et al. (2011) studied gamete nuclear migration in a *C. elegans arp2/3* mutant and showed that Arp2/3-dependent actin nucleation facilitated microtubule growth required for the movement of the male gamete nucleus to join the female gamete nucleus. These results suggest that not only in rodents, but also in *C. elegans*, F-actin plays a role in gamete nuclear migration and the degree of F-actin participation depends on the species. Planarians do not have centrosomes (Azimzadeh et al., 2012) and it would be of interest to know how planarians use microtubules and F-actin in the fertilized egg to control gamete nuclear migration.

## Factors Linking Microtubules and Gamete Nuclei for Migration

It has been proposed that gamete nuclei associate with microtubules through the microtubule motor proteins, dynein, and kinesin. Cytoplasmic dynein was seen to be enriched around the gamete nuclei of the one-cell embryo in *C. elegans* and functional disruption of dynein by RNA-mediated interference (RNAi) resulted in female gamete nuclei of *C. elegans* unable to migrate (Gönczy et al., 1999). A defect in male gamete nuclear migration in *unc-83* (an outer nuclear specific cargo adaptor for kinesin-1) mutant (Xiong et al., 2011) could indicate the role of kinesin for gamete nuclear migration in *C. elegans*. In *Drosophila*, there has been no report of dynein function for gamete nuclear migration. A *kinesin like protein 3A* mutant gene prevented female gamete nuclear migration in *D. melanogaster* (Williams et al., 1997), confirming the requirement of the microtubule motor protein for female gamete nuclear migration.

**FIGURE 3:**
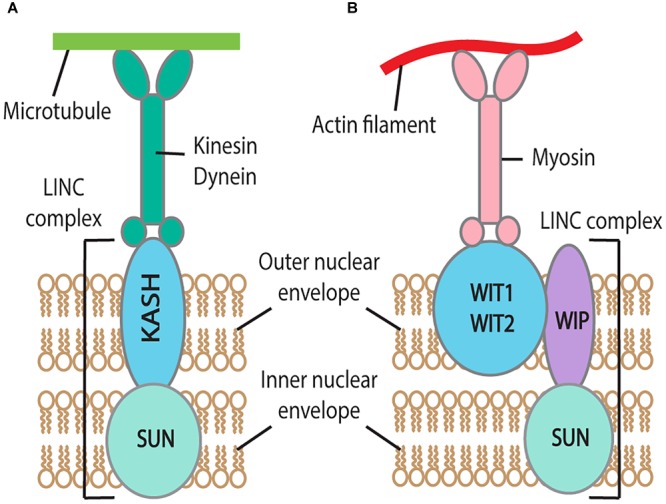
Factors linking cytoskeleton and gamete nuclear envelope for migration in animals **(A)** and its one possible model in flowering plants **(B)**. **(A)** In animals, the LINC complex, containing KASH domain protein in the outer nuclear envelope and SUN in the inner nuclear envelope, links nucleus-kinesin/dynein-microtubules. **(B)** In flowering plants, the LINC complex links nucleus-myosin-actin in somatic cell nuclei and the pollen tube vegetative nucleus. Like animals, the SUN domain protein is present in the inner nuclear envelope in flowering plants that is connected with the outer nuclear envelope embedded proteins, WIT1/2 and WIP.

Factors from the gamete nuclear envelope involved in interaction with microtubules have also been investigated. A Linker of Nucleoskeleton and Cytoskeleton (LINC) complex connects the nuclear envelope to the cytoskeleton (Figure 3A) (Luxton and Starr, 2014). The LINC complex consists of a Klarsicht/ANC-1/Syne Homology (KASH)-domain protein localized in the outer nuclear membrane and a Sad1/UNC-84 (SUN)-domain protein in the inner nuclear membrane. The SUN domain is conserved both in animals and plants (Figure 3A) (Evans et al., 2014). In *C. elegans*, the KASH-domain protein ZYG-12 binds to dynein and both ZYG-12 and the SUN-domain protein SUN-1 are required for gamete nuclear migration (Malone et al., 2003; Minn et al., 2009; Zuela et al., 2016). In zebrafish, the KASH-domain protein lymphoid-restricted membrane protein (Lrmp) was also required for gamete nuclear migration (Lindeman and Pelegri, 2012), suggesting a conserved LINC complex function, linking nucleus-dynein-microtubules for gamete nuclear migration. In *Drosophila*, there are several SUN and KASH domain proteins present; however, they do not play a role in gamete nuclear migration and the detailed mechanism by which *Drosophila* gamete nuclei migrate along microtubules remains unknown (reviewed in Kim et al., 2015). The involvement of the LINC-complex for proper positioning of the nucleus has been proved in the muscle cell; the nucleus anchorage in mouse muscle cell was severely disrupted in *sun* knockout mutant mice (Lei et al., 2009). However, the role of the LINC-complex in actin-dependent gamete nuclear migration in mouse is yet to be discovered.

## Gamete Nuclear Migration in Brown Algae

**FIGURE 4:**
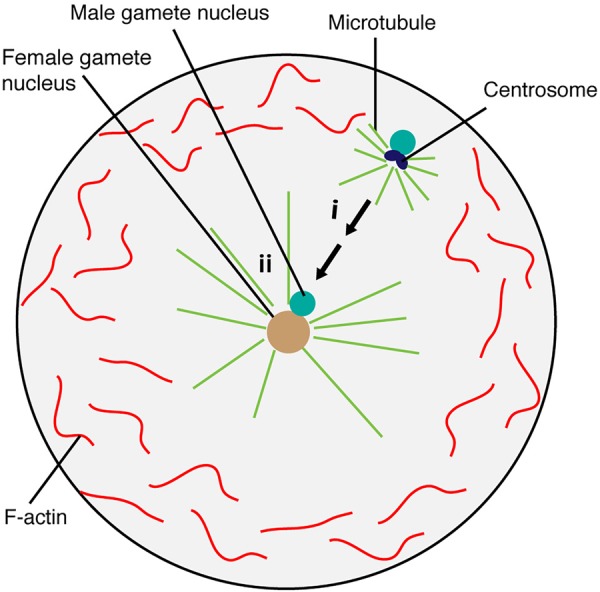
Brown algae sperm nuclear migration toward the egg nucleus. The egg nucleus stays at the center, which is maintained by microtubule and F-actin. Microtubules generate sperm aster around the sperm in the fertilized egg cell (i). Sperm nucleus then moves toward the egg nucleus positioned at the center for fusion (ii).

Historically, brown algae have been intensively studied as model organisms for fertilization because (i) large numbers of gametes can easily be collected for experiments, (ii) many species produce sessile eggs and small motile sperm, performing oogamous fertilization like animals, and (iii) other modes of fertilization such as anisogamy (dissimilar size but identical motility) and isogamy (gametes of similar size and motility) are also present in this group, allowing a wide range of research to understand fertilization (reviewed in Quatrano, 1978). Like in most animals, in the brown alga *Fucu*s *distichus*, paternally derived centrioles establish the centrosome in the fertilized egg cell (Motomura, 1994; Nagasato, 2005), and microtubules then generate sperm asters (Figure 4). The effects of microtubule inhibitor nocodazole, but not F-actin inhibitor cytochalasin D, showed the pivotal role of microtubules in gamete nuclear migration (Supplementary Table 1) (Brawley and Quatrano, 1979; Swope and Kropf, 1993). Interestingly, unlike animals, the egg nucleus stays at the center and the sperm nucleus moves toward the egg nucleus for fertilization in *F. vesiculosus* and *Pelvetia fastigiata* (Figure 4) (Brawley et al., 1976; Swope and Kropf, 1993). Cytoskeleton inhibitor treatments (nocodazole and cytochalasin D) indicated that this centered position of the egg nucleus depends on both microtubules and F-actin (Supplementary Table 1) (Swope and Kropf, 1993). It remains to be solved exactly how microtubules and F-actin control the migration of sperm nuclei in brown algae.

## The Unique Fertilization Processes of Red Algae

**FIGURE 5:**
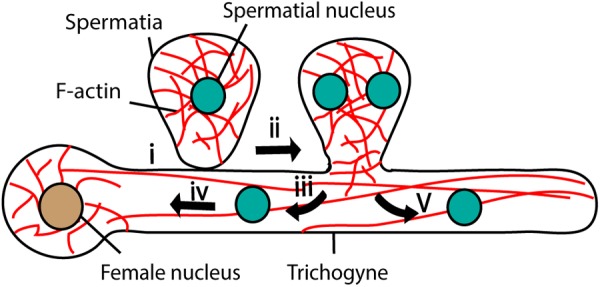
Fertilization in Red algae. Female gamete adheres to the male gamete (spermatia) through trichogyne (i). After adhesion, spermatial nucleus undergoes mitosis (ii) and generates two nuclei; one migrates toward the female nucleus through trichogyne using F-actin (iii and iv) and the other moves toward the tip of the trichogyne (v).

Unlike those of brown algae and animals, red algal male gametes (spermatia) are immotile and their movement relies on aquatic currents and diffusion. The female gamete develops a trichogyne, a hair-like cytoplasmic extension, that adheres to the male gamete (Figure 5) (Pickett-Heaps and West, 1998). After adhesion, the spermatial nucleus undergoes mitosis and generates two nuclei (Figure 5) (Pickett-Heaps and West, 1998). Both nuclei enter the female gamete trichogyne; one moves toward the egg nucleus and the other moves to the opposite direction and stops at the tip of the trichogyne (Figure 5) (Pickett-Heaps and West, 1998).

During migration, there were no obvious microtubule structures detected in the red alga, *Bostrychia moritziana* trichogyne (Wilson et al., 2002). Furthermore, the application of microtubule inhibitors such as oryzalin or griseofulvin did not affect spermatial nuclear migration (Wilson et al., 2002), suggesting that the migration mechanism differs from that of brown algae and animals. F-actin generates a mesh-like arrangement around the spermatial nuclei and long bundles parallel to the longitudinal axis of the trichogyne (Pickett-Heaps and West, 1998; Wilson et al., 2002, 2003). The application of F-actin inhibitors halted spermatial nuclear movement, although the sensitivity varied among species of red algae (Supplementary Table 1) (Kim and Kim, 1999; Wilson et al., 2002). Application of 2,3-butanedione monoxime (BDM), an inhibitor of ATP hydrolysis in the myosin motor domain also arrested sperm nuclear migration (Supplementary Table 1) (Wilson et al., 2002), suggesting the actomyosin control of gamete nuclear migration in red algae.

## Variations of Sexual Reproduction in Green Algae

The most detailed observation of green algae sexual reproduction has been performed on biflagellate *Chlamydomonas*, which contains two isogamous mating types: plus mating type (MT+) and minus mating type (MT-) (reviewed in Mori et al., 2015). Mating is followed by the gamete activation, gamete attachment, and fusion. The activated gametes make a tubular mating structure (TMS) from flagella. TMS varies among isogamous species in green algae. In *C. reinhardtii*, only MT+ make TMS (reviewed in Mori et al., 2015) but in case of *Gonium pectoral*, both MT+ and MT- contribute to the TMS (Mogi et al., 2012). The presence of actin filaments has been visualized in *Chlamydomonas* TMS by an anti-actin immunofluorescence study (Detmers et al., 1985). Moreover, a deletion mutation of an actin gene (*ida5*) resulted in abnormal TMS with poor mating efficiency (Kato-Minoura et al., 1997). Taken together, F-actin plays a role in *Chlamydomonas* mating and further studies to understand the molecular and cellular dynamics of green algae mating are awaited.

*Chlamydomonas* belongs to the Chlorophyta, one of the two clades of green algae, and the other, Charophyta, is evolutionarily linked to land plants. Both clades of green algae show changes in the mode of sexual reproduction from isogamy to anisogamy (reviewed in Mori et al., 2015), and especially the Charophyta display the evolution of sexual reproduction that leads to what is seen in land plants. Very recently, the MYB transcription factor DUO1 has been proposed as the key factor that allowed the evolution of modes of sexual reproduction in the land plant lineage from isogamy to anisogamy in green algae and early diverging land plants to immotile sperm cells in flowering plants (Higo et al., 2018). However, less is known about the molecular and cellular mechanisms by which Charophyta green algae perform gamete nuclear migration.

## Sperm Nuclear Migration in Early Diverging Land Plants

Like animals and brown algae, fertilization in early diverging land plants such as bryophytes and ferns involves the release of flagellated motile male gametes into the environment to swim to non-motile eggs (Lopez-Smith and Renzaglia, 2008); however, the molecular and cellular dynamics of fertilization in bryophytes are not well known. The liverwort, *Marchantia polymorpha*, is dioecious, and therefore, fertilization can be controlled to visualize the process for further study (Shimamura, 2016). Indeed, *Marchantia* has emerged as a model species that can bridge green algae and land plants (Bowman et al., 2017) and understanding of bryophyte fertilization mechanisms will hopefully advance in the near future.

**FIGURE 6:**
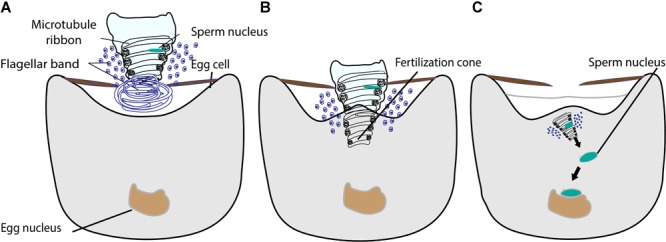
The fusion of male and female gamete in fern. **(A)** The sperm is coiled with an elongated nucleus, a ribbon of microtubules, and a dense layer of flagellar bands. The proximal part of the sperm is illustrated intact and the distal part is shown in cross section. **(B)** The sperm enters into the egg cytoplasm with nucleus, microtubule ribbon, and flagellar band by engulfing. The egg cytoplasm outflows into the region of sperm coil, forming a fertilization cone. **(C)** The sperm chromatin decondenses and moves toward the egg nucleus for fusion.

The fusion of gametes in ferns takes place by an engulfing action; all the components of the male gamete can be found in the female cytoplasm (Figure 6) (Bell, 1975; Myles, 1975, 1978). In the ferns *Pteridium aquilinum* and *Marsilea vestita*, the anterior end of the sperm is coiled, with an elongated nucleus, a ribbon of microtubules, and a dense layer of flagellar bands (Figure 6A) (Bell, 1975; Myles, 1975, 1978). Upon plasma membrane fusion, the egg cytoplasm flows into the region of the sperm coil, forming a fertilization cone (Figure 6B) (Myles, 1978). There were no obvious sperm aster microtubule structures observed with the electron microscope and the sperm nucleus migrates to the egg nucleus for gamete nuclear fusion (Bell, 1975; Myles, 1975, 1978). The application of the microtubule inhibitor colchicine throughout *Marsilea* archegonium development significantly slowed down different steps of fertilization and even inhibited karyogamy (Supplementary Table 1) (Kuligowski et al., 1987). In another study, the addition of colchicine to the culture medium during the final stage of archegonium development affected the ultrastructure of the fertilization cone, through which the engulfed spermatozoid moved toward the female nucleus (Figure 6B,C and Supplementary Table 1). The colchicine treatment caused the fertilization cone to fail to form or to be poorly defined, and the migration of the male nucleus was disturbed and considerably lengthened (Kuligowski et al., 1985). These results suggest that microtubules directly or indirectly play a role in gamete nuclear migration in *Marsilea.* Further investigations of detailed mechanisms of how microtubules participate in gamete nuclear migration in ferns are awaited.

## Gamete Nuclear Migration in Flowering Plants

**FIGURE 7:**
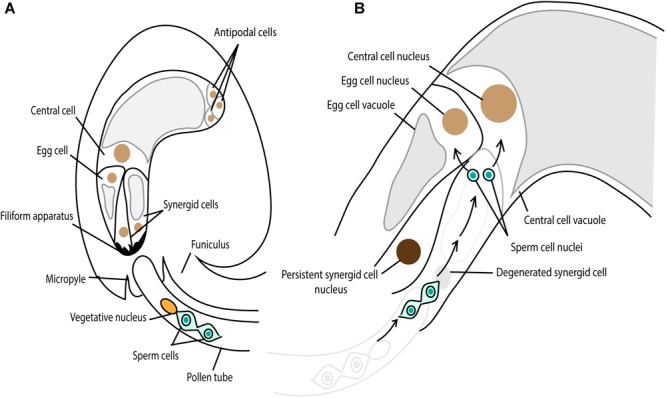
Double fertilization in *Arabidopsis.* **(A)** The pollen tube grows toward the female gametophyte, comprising the egg cell, two synergid cells, the central cell and three antipodal cells. The chemoattractant, secreted from the synergid cell filiform apparatus, guides the pollen tube through the micropylar end. **(B)** One synergid cell receives the pollen tube and the two sperm cells are released from the pollen tube. One sperm cell fertilizes the egg cell, giving rise to embryo and the other one fertilizes the central cell, developing the endosperm.

During the evolution of land plants, the adaptation to land facilitated changes not only in the body plan, such as vascular tissue formation, but also in the mode of sexual reproduction. As noted earlier, early diverging land plants such as liverworts, mosses, and ferns, require exogenous films of water so that the released motile sperm can swim to the egg cell for fertilization (reviewed in Paolillo, 1981). By contrast, seed-bearing plants such as gymnosperms and angiosperms developed the pollen grain and pollen tube by which sperm or sperm cells are delivered proximal to the egg cell without any supply of water from the outside environment for fertilization. In basal gymnosperms such as *Ginkgo* and cycads, the sperm retains flagella that propel them along the pollen tube. In other gymnosperms such as conifers, flagella have been lost but the pollen tube is siphonogamous, able to transfer the sperm cell to the egg cell through the pollen tube (reviewed in Southworth and Cresti, 1997; Rudall and Bateman, 2007; Lora et al., 2016). Sperm cells in flowering plants are also immotile, and have lost components required for centrosome formation (reviewed in Carvalho-Santos et al., 2011). Furthermore, flowering plants perform double fertilization in which two sperm cells existing in one pollen tube fertilize the egg cell and central cell in one ovule (Figure 7A) (reviewed in Raghavan, 2006). After the release of two sperm cells proximal to the egg cell and central cell, plasmogamy, gamete nuclear migration, and karyogamy take place as have been seen in many animals and early-diverging land plants (Figure 7B) (reviewed in Kawashima and Berger, 2011). Do flowering plants control gamete nuclear migration in the same way as animals and early-diverging land plants or did flowering plants alter the mechanism as they changed the mode of sexual reproduction?

## Early Studies Revealed Changes in Cytoskeleton Usage in Fertilization

Cytoskeleton immunofluorescence of dissected ovules before and after fertilization in nun orchid (*Phaius tankervilliae*), *Plumbago zeylanica*, tobacco (*Nicotiana tabacum*), and maize (*Zea mays*) revealed the prominence of F-actin bands at the interface between the egg and central cell where the fusion of male gametes takes place (Huang et al., 1993; Huang and Russell, 1994; Huang and Sheridan, 1998; Ye et al., 2002). However, the detailed structure and dynamics of F-actin in the female gametes during gamete nuclear migration was still missing. This was partly because the female gamete cells are embedded deep inside maternal tissues, hindering the observation of fertilization events and the detection of immuno-labeled signals. To overcome the aforementioned problem, *Torenia fournieri* was first utilized. The egg cell and half of the central cell in *Torenia* protrude from the ovule, enabling the direct observation of fertilization processes (Higashiyama et al., 1997). Using microinjection and confocal laser scanning microscopy, it became evident in *Torenia* that the F-actin cables across the egg cell changed to actin patches during fertilization (Huang et al., 1999; Fu et al., 2000). This result allowed us to speculate that F-actin also plays an important role in fertilization processes, including gamete nuclear migration in flowering plants.

## Time-Lapse Live-Cell Imaging of Plant Gamete Nuclear Migration

In *Arabidopsis*, promoters that activate genes specifically in gamete cells were characterized (central cell, Kinoshita et al., 2004; sperm cell, Ingouff et al., 2007; and egg cell, Sprunck et al., 2012). In addition, methods to observe fertilization events directly from dissected ovules under the microscope have been established (Hamamura et al., 2011; Gooh et al., 2015; Susaki et al., 2017), making it possible to perform time-lapse live-cell imaging to visualize fertilization events in a semi *in vivo* manner using confocal laser scanning microscopy. Hamamura et al. (2011) marked female and male gamete nuclei with fluorescent proteins and visualized the dynamics of gamete nuclei from sperm cell release until karyogamy in *Arabidopsis*. The female gamete nuclei stayed at their positions while two sperm nuclei moved toward the female gamete nuclei for karyogamy (Figure 7B), similar to what has been seen in algae and early-diverging land plants.

## F-Actin Plays Essential Roles in Gamete Nuclear Migration in Flowering Plants

**FIGURE 8:**
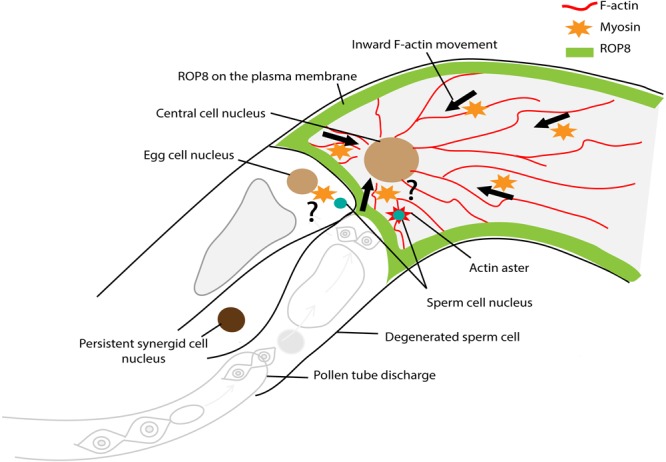
Factors controlling sperm nuclear migration in *Arabidopsis.* F-actin cytoskeleton is involved in sperm nuclear migration in *Arabidopsis*. Upon entry to the central cell, the sperm nucleus becomes surrounded by an aster-shaped F-actin structure. This aster-like structure moves toward the central cell nucleus together with the sperm nucleus for karyogamy. There is a constant “inward” movement of F-actin bundles, from the central cell membrane periphery to the cell nucleus. This inward movement is controlled by plasma membrane associated ROP8 and myosin.

To further understand how the F-actin cytoskeleton was involved in gamete nuclear migration in flowering plants, Kawashima et al. (2014) generated *Arabidopsis* transgenic lines that expressed the lifeact-VENUS fluorescence protein in the egg cell and the central cell. The lifeact peptides specifically bind to F-actin, allowing the visualization of F-actin structures and dynamics in the living cells with minimal artifacts (Riedl et al., 2008; Era et al., 2009). Time-lapse image analysis showed that upon entry to the central cell, the sperm nucleus became surrounded by an aster-shaped F-actin structure and this aster-like structure moved toward the central cell nucleus together with the sperm nucleus for karyogamy (Figure 8). To further understand the requirement of F-actin in gamete nuclear migration in *Arabidopsis*, F-actin cables were specifically disrupted in the female gametes by introducing a semi-dominant negative *ACTIN* transgene (*DN-ACTIN*) (Kato et al., 2010), and the fertilization process was investigated in these transgenic lines (Kawashima et al., 2014). In both the egg cell and the central cell, the expression of *DN-ACTIN* indeed disrupted actin cable structures and arrested the sperm nucleus at the place of gamete fusion, thus, no embryo or endosperm development was initiated (Kawashima et al., 2014). Interestingly, the detailed analysis of central cell F-actin movement showed that prior to fertilization, there was a constant F-actin ‘inward’ movement from the cell membrane periphery to the center of the cell where the nucleus was located (Figure 8) (Kawashima et al., 2014). The movement of the sperm nucleus, as well as the F-actin aster surrounding the sperm nucleus toward the central cell nucleus coincided with the constant ‘inward’ movement of F-actin cables, suggesting that central cell F-actin dynamics were already prepared for sperm nuclear migration. Inward movement could only have been discovered using live cell imaging which offered an additional level of analysis of cytoskeletal processes.

*In vitro* fertilization in rice, maize, and tobacco also allowed us to obtain real-time observations of fertilization events (Faure et al., 1994; Ohnishi et al., 2014; Ohnishi and Okamoto, 2017; Peng et al., 2017). Like in *Arabidopsis*, in *in vitro* fused rice egg cells, F-actin formed a mesh-like structure that converged to the egg nucleus. The sperm nucleus appeared to be carried toward the egg nucleus by the continuous convergence of the actin meshwork (Ohnishi et al., 2014; Ohnishi and Okamoto, 2017). Furthermore, *in vitro* fertilization enables the application of cytoskeleton inhibitors to monitor their effects on fertilization. Ohnishi et al. (2014) showed that the application of the F-actin drug, latrunculin B, stopped sperm nuclear migration, consistent with the result observed in *Arabidopsis* (Kawashima et al., 2014) (Supplementary Table 1). *In vitro* fertilization of tobacco and maize also revealed the importance of F-actin for sperm nuclear migration (Supplementary Table 1) (Peng et al., 2017), demonstrating that F-actin involvement in sperm nuclear migration is conserved throughout flowering plants.

In the *Arabidopsis* central cell, the nucleus is proximal to the egg cell where the sperm cells are released from the pollen tube for plasmogamy. The expression of either *DN-ACTIN* or over-expression of the wild-type *ACTIN* in the central cell shifted the nucleus toward the chalazal end, separating the nucleus away from the egg cell (Kawashima and Berger, 2015). This suggests that F-actin also plays a role in the positioning of the female gamete nucleus that is likely due to the balance of F-actin dynamics. However, fertilization in wild-type *ACTIN* over-expression lines was normal (Kawashima and Berger, 2015). The position of the central cell nucleus proximal to the egg cell likely contributes to successful fertilization, yet, it remains unclear to what degree this plays a role in successful fertilization in flowering plants.

## Microtubules Are Dispensable for Sperm Nuclear Migration in Flowering Plants

In flowering plants, F-actin plays a pivotal role in sperm nuclear migration, but how about microtubules? The applications of microtubule inhibitors such as colchicine and oryzalin, did not show any effect on sperm nuclear migration in *in vitro* fertilization of rice, tobacco, and maize (Supplementary Table 1) (Ohnishi et al., 2014; Peng et al., 2017). In order to confirm the involvement of microtubules in sperm nuclear migration *in planta*, Kawashima et al. (2014) utilized the *Arabidopsi*s *tubulin folding cofactor c* mutant (*por*). The *por* mutant and other cofactor mutants showed seed abortion in *Arabidopsis* (Steinborn et al., 2002); however, whether it was fertilization itself that failed in these mutants remained unclear. Using a microtubule fluorescent marker, half of the *por/+* female gametophytes showed no microtubule structures, confirming that the *por* female gametophytes did not generate microtubules. The sperm chromatin marker showed successful diffusion of the male chromatin into female gamete nuclei in almost all ovules when the *por/+* heterozygous mutant was self-pollinated (Kawashima et al., 2014). Taken together, these results demonstrate that microtubules are dispensable for sperm nuclear migration. The structure and dynamics of F-actin in the *por* female gametes remained normal (Kawashima et al., 2014), confirming that F-actin is the essential component for sperm nuclear migration.

## Rop Pathway Controls the Constant Inward F-Actin Movement in the Female Gametes

As the movement of F-actin in the female gamete was from the membrane periphery to the center of the cell (Kawashima et al., 2014; Ohnishi and Okamoto, 2017), the membrane-associated proteins involved in F-actin assembly were further investigated. Plant-specific Rho-GTPases (ROPs) are well-characterized factors that can associate with the plasma membrane and control F-actin dynamics through a ROP signaling cascade (reviewed in Craddock et al., 2012). Among those factors, the *Arabidopsis* transcriptional profiling covering from gamete cells (sperm, egg, and central cell) to individual compartments (the embryo, endosperm, and seed coat) of the seed throughout development (Borges et al., 2008; Le et al., 2010; Wuest et al., 2010; Belmonte et al., 2013) identified that *ROP8* was specifically expressed in the central cell-lineage (i.e., the central cell and endosperm) (Kawashima et al., 2014). Indeed, both transcriptional and translational fusion constructs of *ROP8* showed central cell-specific expression and plasma membrane association (Figure 8) (Kawashima et al., 2014). To investigate the function of ROP8 in F-actin inward movement, dominant-negative (DN) and constitutively-active (CA) forms of ROP8 were expressed in the central cell by the native *ROP8* promoter. When the balance of the ROP activity within a cell is essential for its proper function, both the DN and CA mutations of ROP show developmental defects; *DN-ROP1* in the pollen inhibited polar growth, whereas *CA-ROP1* induced non-polarized isotropic growth in the pollen tube in *Arabidopsis* (Li et al., 1999). By contrast, *CA-ROP8* showed F-actin dynamics and sperm nuclear migration similar to wild type, whereas, *DN-ROP8* showed reduced inward F-actin movement along with halted sperm nuclear migration in the *Arabidopsis* central cell (Kawashima et al., 2014). These results suggest that ROP8 might be constantly active in the entire central cell plasma membrane and maintain the constant inward movement of F-actin prior to fertilization in the central cell.

In both animals and plants, small Rho GTPase pathways promote F-actin nucleation via direct interactions with formins (Kovar et al., 2006) or with Wiskott–Aldrich syndrome protein (WASP), neural (N)-WASP and WASP family verprolin-homologous (WAVE, also named SCAR) proteins that relay activation signals from small GTPases to the actin-nucleating Arp2/3 complex (Millard et al., 2004; Basu et al., 2008; Yalovsky et al., 2008). Upon activation, the Arp2/3 complex contributes to the ATP-dependent actin nucleation (Welch et al., 1997, 1998). The application of wiskostatin, a cell-permeable chemical inhibitor of WASP, halted the migration of the sperm nucleus toward the egg nucleus during *in vitro* fertilization of tobacco and maize (Peng et al., 2017). This result suggests that the ROP-WASP-ARP2/3 complex signal cascade controls the inward dynamics of F-actin in the female gamete of flowering plants. However, mutants of ARP2/3 complex genes in *Arabidopsis* were fertile (Mathur et al., 2003). Further analyses are required to understand whether the ARP2/3 complex plays a role in female gamete F-actin inward movement as well as whether there are differences among species or between the egg cell and central cell.

## The Possible Involvement of Myosin in Sperm Nuclear Migration in Flowering Plants

Actin can generate organelle motility in association with the motor protein myosin. The application of BDM disorganized the F-actin structure in pollen tubes and short root hair cells of *Hydrocharis dubia* (Supplementary Table 1) (Tominaga et al., 2000). In *Arabidopsis*, myosin knockout mutants showed disorganization of F-actin bundles in root hair cells (Ueda et al., 2010). Indeed as noted earlier, mouse gamete nuclear migration and male gamete nuclear migration in red algae are myosin dependent (Wilson et al., 2002; Chaigne et al., 2016). Is myosin involved in the constant inward F-actin movement in the female gamete of flowering plants? BDM application to the *Arabidopsis* female ovule impaired the inward F-actin movement in the central cell (Supplementary Table 1) (Kawashima et al., 2014). In addition, during *in vitro* fertilization of rice, the application of BDM not only impaired F-actin actin movement but also arrested sperm nuclear migration toward the egg nucleus (Supplementary Table 1) (Ohnishi and Okamoto, 2017). These results suggest that myosin is involved in the constant inward movement of F-actin in the female gamete of flowering plants (Figure 8). However, BDM showed no effect on sperm nuclear migration in tobacco and maize *in vitro* fertilization (Supplementary Table 1) (Peng et al., 2017). It is noteworthy that the concentration of applied BDM differs among experiments (Supplementary Table 1). Nevertheless, all results so far have been based only on drug applications. As in red algae (Kim and Kim, 1999; Wilson et al., 2002), sensitivity to chemical inhibitors might vary among species. Molecular and/or genetic evidence to verify the involvement of myosin in F-actin dynamics in sperm nuclear migration is awaited.

The link between F-actin and gamete nuclei in flowering plants are still unknown. Like dynein with microtubules in *C. elegans* (Gönczy et al., 1999; Simone et al., 2018), the association of myosin XI-I with the nuclear envelope of somatic cells has also been reported in *Arabidopsis* (Tamura et al., 2013). In this study, co-immunoprecipitation followed by mass spectrometry identified that Myosin XI-I directly interacts with outer nuclear membrane proteins such as the tryptophan-proline-proline (WPP) domain interacting tail-anchored protein1 (WIT1) and WIT2 (Figure 3B). The bidirectional nuclear movement in the root hair of the myosin *xi-i* mutant was dramatically slowed compared to wild type (Tamura et al., 2013). WITs are also essential to pollen vegetative nuclear migration; loss-of-function mutations in *WIT* and/or WPP domain interacting protein (*WIP)* gene families resulted in impaired vegetative nucleus movement in the *Arabidopsis* pollen tube (Zhou and Meier, 2014). These results show that myosin can directly bridge F-actin and the nucleus, raising the possibility that myosin directly links the sperm nucleus with the female gamete F-actin for migration.

## Concluding Remarks

Many findings on gamete nuclear migration in flowering plants have been recently accomplished, yet there are many more questions raised. Are there differences in the mechanisms among flowering plant species? How about between the egg cell and central cell? ROP8 is specific to the central cell (Kawashima et al., 2014) and there should be other factors expressed in the egg cell controlling F-actin dynamics. Calcium signaling might also be involved in the speed of F-actin inward movement during sperm gamete nuclear migration (Hamamura et al., 2014; Ohnishi and Okamoto, 2017). We are just at the start of the journey to understand the molecular details of plant gamete nuclear migration. Behind those details are questions about why differences evolved. Perhaps, the more details we uncover in different species, the clearer the reasons for their evolution will become.

The advancement in live-cell imaging technologies and techniques has allowed us to further investigate the molecular and cellular mechanisms of plant reproduction dynamics as a continuous process. Cytoskeleton dynamics in the female gametophyte during fertilization and the onset of embryo development has been recently revealed: the dynamics of microtubules and F-actin have been investigated during zygote polarization in *Arabidopsis* (Kimata et al., 2016) as well as during the unique synergid-endosperm cell–cell fusion (Maruyama et al., 2015; Motomura et al., 2018). From gamete cell fusion, sperm nuclear migration, gamete nuclear fusion, initiation of embryo and endosperm development, to synergid-endosperm fusion, these individual developmental processes are tightly linked to each other such that these highly orchestrated processes successfully initiate seed development. Cytoskeletons control complex cellular dynamics and it is found that flowering plants have evolved a distinct mechanism, in which F-actin, instead of microtubules, plays the essential role (reviewed in Tominaga and Ito, 2015; Davidson and Wood, 2016; Paez-Garcia et al., 2018). Further investigation of gamete nuclear migration, together with pre- and post-fertilization processes will not only facilitate our knowledge of the dynamic and complex process of plant reproduction but also help gain insights into plant cytoskeleton biology as well as the evolution of plant reproduction.

## Author Contributions

UF and TK designed the concept of the manuscript. UF, MA, ZH, and TK wrote the manuscript. AC and TK edited the manuscript.

## Conflict of Interest Statement

The authors declare that the research was conducted in the absence of any commercial or financial relationships that could be construed as a potential conflict of interest.
